# La alianza entre el fabricante de IVD y el laboratorio clínico en el control de calidad de los resultados

**DOI:** 10.1515/almed-2020-0107

**Published:** 2020-12-07

**Authors:** Marco Pradella

**Affiliations:** Sociedad Italiana de Patología Clínica y Medicina de Laboratorio, SIPMeL, Castelfranco Veneto, Italia

**Keywords:** Acreditaciones, control de calidad, ISO 15189, ISO 15198

Estimado Editor,

La norma ISO 15189, actualmente en fase de desarrollo [1], supone tener que reevaluar el rendimiento de los métodos, esto es, realizar un control de calidad (QC), tal como se define en la sección 3.3.7 de la norma ISO 9000 [2].

La Comisión de Calidad y Acreditaciones de la Sociedad Italiana de Patología Clínica y Medicina de Laboratorio (SIPMeL) emitió sus recomendaciones sobre la materia [3], haciéndose eco del documento ISO 15198:2004 [4], confirmado por primera vez en 2008 y revisado con resultados favorables en diciembre de 2018. Sus aspectos clave son: la responsabilidad compartida entre el laboratorio y el fabricante, el uso de términos y definiciones específicas, y la validación y revalidación de los procedimientos de control de calidad actualizados.

La ISO 15198 está incluida en el paquete ISO para laboratorios clínicos, ha sido aceptada y revalidada en los documentos ISO actuales o en fase de desarrollo, así como en multitud de documentos del Instituto de Estándares del Laboratorio Clínico (CLSI) y en algunos documentos de la Organización Mundial de la Salud (OMS) [3].

La ISO 15198 revierte una práctica común muy extendida entre los laboratorios clínicos. La responsabilidad sobre el QC es compartida por los fabricantes y los usuarios de dispositivos médicos de diagnóstico *in vitro* (IVDR). El mismo principio rige la norma ISO/TS 22367 (gestión de riesgos) [5], [ 6], así como varios puntos de la ISO 15189. Reforzar la colaboración entre el laboratorio y el fabricante se encuentra entre los principales objetivos del llamado “Plan individualizado de control de calidad”, introducido en los EE.UU. como alternativa a las estrictas normas contenidas en las “Enmiendas de mejora del laboratorio clínico” [7].

La ISO 15198 incorpora términos ya definidos en varias ISO y estándares europeos (EN), aunque añade nuevas definiciones: 3.5 procedimientos de control, 3.7 procedimientos de examen, 3.11 condiciones de precisión intermedia, 3.12 lote.

El QC se suele realizar incorrectamente en los laboratorios. Una encuesta sobre prácticas de control de calidad en los laboratorios [8] describe el uso habitual de la norma de control 1_2s_ en la mayoría de los laboratorios. En la llamada “Gran encuesta de QC 2017” realizada por Westgard [9], se observó que el 55% de los laboratorios aplican la norma de control 1_2s_ en todos los test. En la mitad de los laboratorios, el control únicamente se examina una vez al día, antes de empezar a procesar las muestras de los pacientes. Además, casi la totalidad de los laboratorios emplean materiales de control preparados por los fabricantes del sistema, o por terceros, con o sin valor asignado. Una encuesta realizada en Corea entre pequeños laboratorios [10] reveló que el control interno de calidad se ve obstaculizado por varios factores, principalmente el coste, la falta de personal o de tiempo, y dificultades operativas debido a la falta de competencias o de recursos informáticos.

Según los procedimientos de QC del punto 4.1 y el análisis de riesgos del punto 4.2 de la norma ISO 15198, el fabricante deberá especificar en las instrucciones de uso cuáles son los materiales de control válidos, indicar la forma de establecer los criterios para evaluar la validez del procedimiento de medición, y ofrecer recomendaciones sobre los pasos a dar en caso de que se obtengan resultados de control de calidad inaceptables. Además, deberá proporcionar información suficiente para explicar los principios en los que se sustentan las instrucciones.

Los procedimientos de QC incluyen un método de detección (p.ej. material de control de calidad, un sistema de monitorización electrónico o un control químico interno) así como los criterios de adecuación que determinarán la presencia de un error crítico. Se identificarán y describirán las limitaciones del procedimiento de QC. El método de análisis de riesgos deberá tener en cuenta el uso al que está destinado el dispositivo, así como las necesidades del laboratorio. Además, se identificarán las fuentes de variabilidad y posibles riesgos que ni el diseño del dispositivo ni los controles del proceso de fabricación pueden mitigar.

La ISO 15198 exige la validación de los procedimientos de control de calidad (puntos del 5.1 al 5.5), así como su monitorización y revalidación (punto 5.6). El protocolo de validación deberá incluir los test actuales y/o simulados en condiciones de error. El fabricante comprobará periódicamente la adecuación de los procedimientos de control de calidad recomendados, especialmente cuando se efectúen cambios en el diseño del dispositivo o se comuniquen eventos adversos.

Mejorar el papel del fabricante del dispositivo diagnóstico ofrece múltiples ventajas: aporta recursos materiales e información a los laboratorios clínicos, cuyos recursos para este tipo de actividades no son siempre los adecuados, ya que estas suelen ser complejas y costosas; favorece la armonización de los procedimientos entre diferentes laboratorios, y permite comparar la calidad de los servicios suministrados. Así mismo, permite la identificación de aspectos críticos concretos que, una vez comunicados al fabricante y comparados con los identificados en otros laboratorios, puede contribuir a dar con una solución adecuada. De este modo, la SIPMeL recomienda que todos los fabricantes de dispositivos diagnósticos *in vitro* proporcionen a los laboratorios instrucciones de control interno de calidad.
